# DSA-based perfusion parameters *versus* TICI score after mechanical thrombectomy in acute ischaemic stroke patients: a congruence analysis

**DOI:** 10.1186/s41747-024-00534-1

**Published:** 2024-12-05

**Authors:** Sebastian R. Reder, Andrea Kronfeld, Sonja Gröschel, Arda Civelek, Klaus Gröschel, Marc A. Brockmann, Timo Uphaus, Marianne Hahn, Carolin Brockmann, Ahmed E. Othman

**Affiliations:** 1 Department of Neuroradiology, University Medical Center, Johannes Gutenberg-University of Mainz, Mainz, Germany; 2 Department of Neurology, University Medical Center of the Johannes Gutenberg-University of Mainz, Mainz, Germany

**Keywords:** Angiography (digital subtraction), Ischemic stroke, Outcome, Perfusion imaging, Thrombectomy

## Abstract

**Background:**

Several factors are frequently considered for outcome prediction rin stroke patients. We assessed the value of digital subtraction angiography (DSA)-based brain perfusion measurements after mechanical thrombectomy (MT) for outcome prediction in acute ischaemic stroke.

**Methods:**

From DSA image data (*n* = 90; 38 females; age 73.3 ± 13.1 years [mean ± standard deviation]), time-contrast agent (CA) concentration curves were acquired, and maximum slope (MS), time to peak (TTP), and maximum CA concentration (CA_max_) were calculated using an arterial input function. This data was used to predict neurological deficits at 24 h and upon discharge by using multiple regression analysis; the predictive capability was compared with the predictive power of the “Thrombolysis in cerebral infarction” (TICI) score. Intraclass correlation coefficients (ICC) of the NIHSS values were analysed.

**Results:**

The comparison of means revealed a linear trend after stratification into TICI classes for CA_max_ (TICI 0: 0.07 ± 0.02 a.u. to TICI 3: 0.22 ± 0.07 a.u.; *p* < 0.001), and for MS (TICI 0: 0.04 ± 0.01 a.u./s to TICI 3: 0.12 ± 0.0  a.u./s; *p* < 0.001). Regression analyses demonstrated equivalent capabilities for estimating neurological deficits after 24 h and at discharge using both the TICI score and DSA-based perfusion parameters (Δ*R*² ~ 0.03). Compared to the actual NIHSS, the ICC ranged from 0.55 to 0.84 for DSA-based models and from 0.6 to 0.82 for TICI-based models.

**Conclusion:**

Semi-quantitative evaluation of DSA-based perfusion parameters prior to and after MT is feasible and could enhance the objectivity and comparability of MT outcome prediction. This technique may offer novel approaches in acute ischaemic stroke management and data comparability.

**Relevance statement:**

DSA-based brain perfusion measurements following interventional stroke therapy could allow for an experience-independent assessment of reperfusion success. It demonstrates predictive power at least equivalent to the established methods. This could support a future automated DSA-based brain perfusion measurement method.

**Key Points:**

- Currently, the evaluation of stroke therapy success is based on the treating physician’s experience.
- The present study introduces an objective semi-quantitative evaluation method.
- In predicting clinical outcomes, the traditional expert-based and semi-quantitative methods are equivalent.

**Graphical Abstract:**

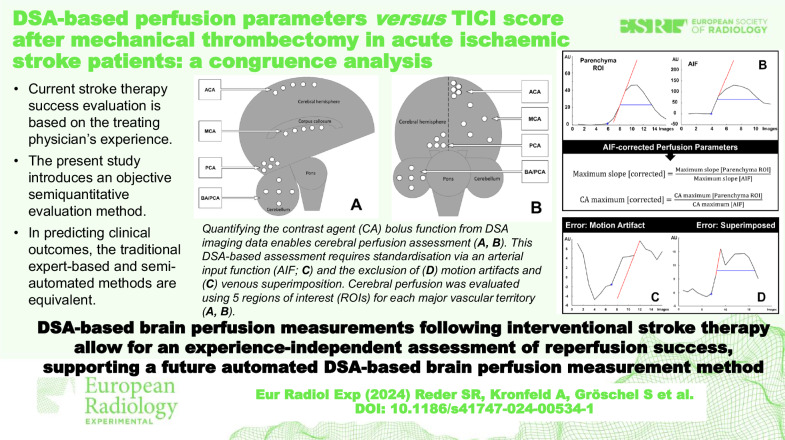

## Background

Stroke is the second most prevalent cause of death worldwide and the leading cause of adult disability [1, 2]. Digital subtraction angiography (DSA)-based mechanical thrombectomy (MT) is the standard therapy for acute ischaemic stroke (AIS) caused by large vessel occlusion [3, 4]. The extent of reperfusion represents a robust predictive determinant for functional independence subsequent to MT [5], traditionally assessed by the semi-quantitative thrombolysis in cerebral infarction (TICI) scale, from 0 indicating no perfusion to 3 indicating complete perfusion [6], or the refined “modified TICI scale”, dividing TICI 2 score into 2a, 2b, and 2c [6].

To obtain a TICI score, the neurointerventionalist subjectively assesses the degree of reperfusion using standardised thresholds defining the degree of reperfusion on the basis of contrast agent (CA) flow following MT. Successful reperfusion is usually defined by achieving a degree of TICI score 2b or higher, aligning with a favourable clinical outcome [7]. With degrees of TICI scores 2b and higher, it becomes increasingly difficult to determine significant differences in correlation with clinical outcomes, suggesting a greater overlap [8]. Regarding expert-based scoring, interrater variabilities become apparent especially when: (i) the experience of neurointerventionalists differs [9–12]; (ii) distal vascular territories are treated [12]; or (iii) partial reperfusion results are achieved [9, 13]. Cohen’s κ of the TICI score evaluation typically ranges between 0.36 and 0.82 [14].

Recent reviews have reported a fluctuating discrepancy between cerebral hyperperfusion and clinical outcomes, suggesting yet unrecognised mechanisms and unconsidered variables [15, 16]. Explaining a poor clinical outcome solely through hypoperfusion after MT may not be sufficient [15]. However, only a few studies compared the validity of DSA images with expert-based assessments focusing solely on either individual large vessel territories (*e.g.*, the middle cerebral artery [MCA]), specific reperfusion grades (*e.g*., TICI 2a or 2b), or a limited number of patients (*e.g*., less than 30) [14, 17–20]. Outcome prediction measurements were not undertaken in these studies.

Consequently, developing methods to objectively quantify cerebral perfusion after MT, independent of the expertise of the neurointerventionalist, is necessary and addressed by recent studies [21, 22]. Preliminary feasibility studies have already demonstrated that two-dimensional imaging datasets resulting from DSA could be used for deriving perfusion data for objective assessment [23–25]. We hypothesised that the extraction of perfusion parameters from DSA data was feasible and could enhance the assessment of reperfusion success after MT, as well as improve the predictive accuracy in estimating changes in neurological deficits compared to the traditional semiquantitative TICI grading. Therefore, we characterised DSA-based perfusion parameters in patients treated with MT to establish an objective approach for evaluating treatment outcomes. The aim was to minimise interindividual differences in assessment, hereby objectifying reperfusion outcomes.

## Methods

### Study design, study population and selection criteria

Patients > 18-years-old (criterion #1) and diagnosed with AIS due to vessel occlusion of the first segments of the anterior cerebral artery (ACA), MCA, or posterior cerebral artery (PCA), *i.e*., A1, first segment of the middle cerebral artery (M1), and first segment of the posterior cerebral artery (P1), respectively (1), admitted to the University Medical Center of the Johannes Gutenberg University Mainz between 2020 and 2022 (criterion #2) were included to the independent, prospective and observational Gutenberg-Stroke-Study (basic parameters including age, sex, body mass index, baseline medication, cardiovascular risk factors, and preexisting conditions). The Gutenberg-Stroke-Study was approved by the responsible ethics committee of the Landesärztekammer Rheinland-Pfalz (approval number: 2018-13335-Epidemiologie). The Gutenberg-Stroke-Study is registered in the German Clinical Trial Registry (DRKS00017253). The DSA image acquisition was conducted as part of the clinical routine, and the evaluation was performed retrospectively. Furthermore, to ensure homogeneity in interindividual blood flow conditions, patients who had intravenous thrombolysis (criterion #3) received the intention to be treated with MT (criterion #4), without combined and/or distant occlusion patterns (> A1 or > M1 or > P1) (criterion #5), without adverse events during hospitalisation (criterion #6), and without a history of prior strokes were selected for analyses (criterion #7). Baseline population characteristics, comorbidities, procedural treatment information and neurological deficits (including adverse events) were acquired. Written informed consent was obtained from all participants or guardians of participants.

The parameter used to assess the success of AIS treatment was the National Institutes of Health Stroke Scale (NIHSS) at 24 h after MT (NIHSS_24h_), at discharge (NIHSS_dc_), and the NIHSS at admission as baseline (NIHSS_adm_). Each of the 12 aspects of the NIHSS score was rated according to guidelines [26–28], resulting in a higher NIHSS score, the more severe the neurological deficit (maximum of 42 points). The DSA perfusion measurement was performed using 90 data sets.

### Angiography setting and TICI score determination

MT was performed on a biplanar angiography unit (Allura Xper FD20, Philips Healthcare, Amsterdam, The Netherlands) and conducted by experienced neuroradiologists (more than five years of cerebrovascular angiography proficiency) using a combined vacuum-locked aspiration-assisted stent retriever technique, with device selection left at the discretion of the interventionalist [29, 30]. Treatment adhered to the standardised “cerebrovascular” DSA protocol of the Angiography Unit (two frames per second, 80 kVp, and activated automatic dose rate control with a weight-adjusted amperage of at least 15 mAs). An iodine-based CA (Ultravist® 300, 300 mg I/mL, Bayer Vital, Leverkusen, Germany) was employed with a 9:1 mixture ratio (9 mL CA; 1 mL 0.9% sodium chloride) and applied at an average rate of 5 mL/s as a bolus injection. The TICI score subjectively classifies brain perfusion after MT as follows: 0 (no perfusion), 1 (minimal flow in distal segments), 2a (< 50% territory perfusion), 2b (≥ 50% territory perfusion), and 3 (complete revascularisation), and was determined by a neurointerventionalist with more than 5 of experience conducting the procedure [31].

### Bolus tracking model and perfusion parameters

Using MATLAB R2022b (The MathWorks, Inc., Natick, MA, USA), an algorithm based on the time-dependent changes in contrasting the brain parenchyma, *i.e*., the C(t)-curve, was developed to analyse the DSA data within predefined regions of interest (ROIs) [23]. In the anterior–posterior and lateral DSA projections, in each of the major vessel territories five ROI were set in the brain parenchyma (diameter of 40 pixels) of the ACA, MCA, or PCA). Due to the limited number of cases with primarily occluded PCA, these were internalised within the basilar artery (BA)/PCA category, hereinafter defined as the “PCA” category. Notably, larger cortical arteries and veins were excluded from the ROI in order to prevent potential inaccuracies in perfusion values. Physiopathological relevance was not considered in favour of achieving a perfusion measurement with minimal overlap, excluding large venous sinuses and adjacent large vessel territories.

**Fig. 1:**
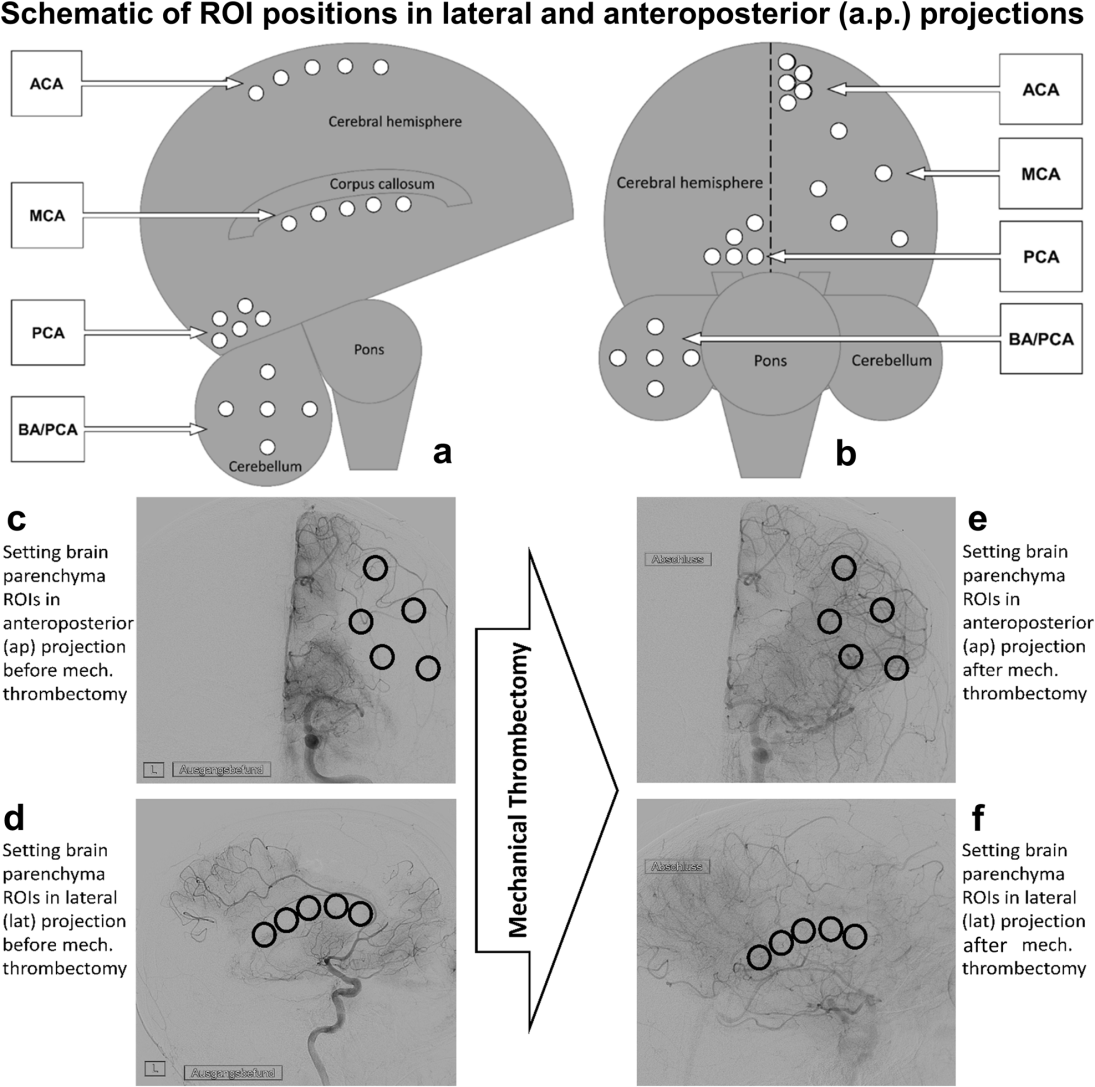
Anatomical landmarks in DSA. In the lateral projection (**a**) for the ACA measurement regions of interest (ROIs) were set immediately below the superior sagittal sinus (SSS), for the MCA below the callosal corpus and for the PCA trapezoidal in projection on the cuneus above the cerebellar tentorium. In the case of the occluded BA and preserved posterior communicating artery (PCOM), the ROI was set in projection on one cerebellar hemisphere. In the anterior–posterior projection (**b**) the ROI for the ACA was set trapezoidal in projection on the apical territory below the SSS, for MCA trapezoidal in projection on the territory and for PCA triangular on the precuneus (or one cerebellar hemisphere in case of preserved PCOM). **c**, **d** Present an occlusion in the MCA territory in anterior–posterior and lateral projections and the setting of the ROI 1 to 5. **e**, **f** Depict the reperfusion result after MT in anterior–posterior and lateral projection

In lateral projection, there were several territory-specific landmarks (Fig. 1a):

– ACA, in line along the apex of the cerebral hemispheres directly below the *superior sagittal sinus*.
– MCA, in line directly below the *callosal corpus*.
– PCA only, trapezoidal, on the *cuneus* directly above the *cerebellar tentorium*;
– BA with subsequent PCA occlusion based on a BA occlusion with the hypoplastic posterior communicating artery (“asteroid” in projection on one cerebellar hemisphere).

In anterior–posterior projection, the ROI was set as follows (Fig. 1b):

– ACA, trapezoidal (apical territory directly below the *superior sagittal sinus* with the broad side along the interhemispheric fissure).
– MCA, trapezoidal (with the broad side along the hemispheric circumference);
– PCA (only), triangular (on the *precuneus*, directly adjacent to the interhemispheric fissure).
– BA/PCA, based on a BA occlusion with the hypoplastic posterior communicating artery (“asteroid” in projection on one cerebellar hemisphere).

**Fig. 2:**
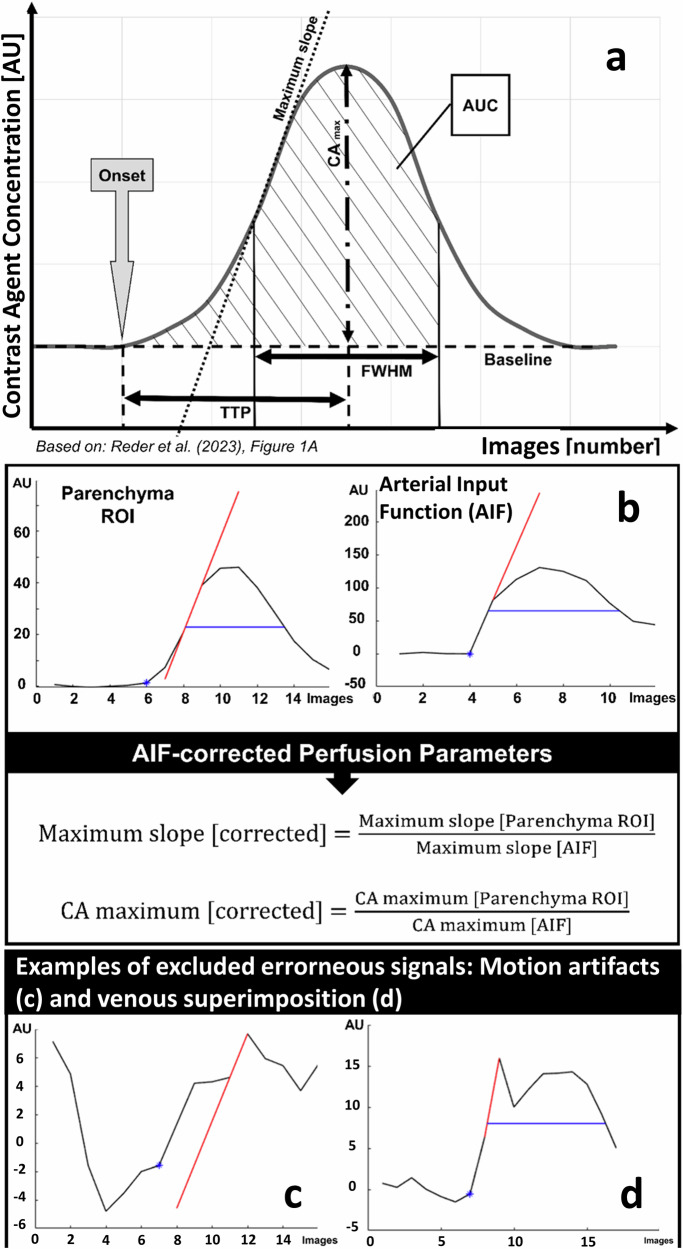
In the MS-based perfusion model (**a**), time-concentration C(t)-curves were determined in the brain parenchyma and as an AIF in the ipsilateral influx vessel. The maximum for the brain parenchyma ranged from about 10 a.u. to 50 a.u., while those for the AIF were approximately 120 to 200. Subsequently, a standardisation algorithm (**b**) was applied. Time-concentration curves affected by motion artefacts (**c**) or overlapping veins (**d**) were excluded

To generate C(t)-curves, the radiation attenuation of each ROI was adjusted by subtracting the baseline signal intensity. Each parameter was standardised by a specific arterial input function (AIF). To measure the AIF, an ROI with a diameter of 150 pixels was set in projection on the cavernous segment of the intracranial internal carotid artery (respectively the middle V3 segment of the vertebral artery). The AIF was determined using 10 pixels within the AIF-ROI with the earliest and most distinct grey value changes. By this, a standardised curve function representing the CA bolus passage (or C(t)-curve; Fig. 2a, b) was obtained. Multiple parameters defined the C(t)-curve, as reported by Reder et al [23]:

– Onset (in s) beginning of the C(t)-curve;
– Difference between the baseline and the maximum of the CA concentration (CA_max_) (in arbitrary units, a.u.): the difference between the baseline and the maximum CA concentration (see Eq. 1);
– Δ*t*_max_, time interval from the onset to the CA_max_;
– time to peak (TTP) (in s), the time interval between the onset and the CA_max_;
– maximum slope (MS) (in a.u./s), point of steepest ascent of the C(t)–curve (see Eq. 2)
– full width of half maximum (FWHM) (in s), the time interval between the MS of the inflowing and outflowing CA bolus;
– area under the curve (AUC), an integral function of the C(t)-curve within the time interval from the onset to the end of FWHM.

AUC and FWHM could not be reliably calculated due to the commonly incomplete acquisition of the venous phase, often constrained by radiation protection measures, and thus were not included in the analysis. The remaining perfusion parameters of the parenchyma ROI were subsequently normalised by dividing each by its corresponding AIF value (Fig. 2b):

1 $${{\rm{Contrast}}}\; {{\rm{agent}}}\; {{\rm{maximum}}}\;{{\rm{CA}}}_{{\rm{max}}}=\frac{{{CA}}_{{{\max}}\,{{\rm{ROI}}}}}{{{CA}}_{{{\max}}\,{{\rm{AIF}}}}}$$

2 $${{\rm{Maximum}}}\; {{\rm{slope}}}\left({MS}\right)=\frac{{{\rm{MS}}}_{{\rm{ROI}}}}{{{\rm{MS}}}_{{\rm{AIF}}}}\;{{\rm{or}}}\;{{{\rm{CA}}}_{{{\max}}}/\Delta t}_{{{\max}}}=\frac{{{{\rm{CA}}}{{\max}} /\Delta t}_{{{\max}}\, {{\rm{ROI}}}}}{{{{\rm{CA}}}{{\max}} /\Delta t}_{{{\max}}\, {AIF}}}$$

In all cases, the C(t)-curve was not acquired until the end of the venous CA washout. Only the arterial and capillary CA phases were used for perfusion measurement (*n* = 90). Consequently, a reliable assessment of an accurate AUC or FWHM for the study population (*n* = 90) was not feasible. Following visual control by a reader, erroneous C(t)-curves due to motion artefacts or signals superimposed by venous contrast were reevaluated (Fig. 2c, d). Patients were excluded from further analyses if irregular artefacts (usually due to motion) exceeded approximately 5 a.u. (see Fig. 2c). Overlaps caused by venous signals were typically identified by a second delayed contrast curve (Fig. 2d). These patients were retained in the analysis; however, alternative areas in the immediate vicinity were sought where the signal was not venously superimposed. For the present study, the primary focus was placed on the analysis of the most resistant parameters: MS, TTP, and CA_max_.

### Statistical analysis

The data was analysed using the statistical software SPSS (version 29, IBM Corp., Armonk, NY, USA). The intraclass correlations (ICC) of the data obtained from the five ROIs (for MS, TTP and CA_max_, each in anterior–posterior and lateral projection) were examined by using reliability analyses (two-way, mixed model; absolute agreement conservative type; 95% confidence intervals) and depicted in matrix scatter plots. Furthermore, non-parametric tests were used (two-sided asymptotic significance level unless declared otherwise; α = 0.05) to compare the mean values of the five ROI (MS, CA_max_, and MTT), and to objectify the differences in the perfusion parameters used between the five ROIs.

The initial TICI score was obtained from the findings of the respective interventionalists. These were re-evaluated by one reader with ten years of experience in evaluating DSA imaging. These evaluations were compared using a weighted Cohen κ (linear weighting). However, the official TICI scores from the radiological reports were used in the analyses.

To compare the perfusion parameters before and after MT and to assess the ability of the method to detect differences, the Friedman test for related samples (a nonparametric test for detecting differences in medians across multiple related groups) was applied.

After stratifying for TICI classes, the Kruskal–Wallis test was used to highlight differences in the means of the perfusion parameters. The differences between the mean values were clustered and analysed according to the affected brain vessel territories (ACA, MCA, and BA/PCA) by using the χ^2^ test. For the exploratory data analysis, principal component analyses were conducted with a rotated component matrix (Rotation method, Varimax with Kaiser-normalisation) to determine potential regression variables. Using perfusion parameters and basic population variables with the highest load on identified subgroups, multiple linear regression analyses with backward elimination and stepwise variable selection were used to predict neurological deficits at 24 h after admission (NIHSS_24h_) and at discharge (NIHSS_dc_) (F probability of inclusion is set at *p* ≤ 0.05, and the probability of exclusion is set at *p* ≥ 0.07) comparing TICI based models to perfusion parameter based ones. In this process, the regression coefficients (R, B, and standardised β; including *p*-values, the determination coefficient *R*²) were provided. Only regression models with predictors with statistically significant influences on the regression equations were reported (*p* < 0.05). For a statistical power of 1 - β = 0.9 with a *R*² = 0.6, an α level of 0.05 and three predictors for multivariate regression analysis the collective should contain *n* = 15 subjects at minimum [32].

Subsequently, the derived regression equations were evaluated for their congruence with the actual NIHSS_24h_ and NIHSS_dc_. These values were reported along with the calculated standard deviations across the entire regression equations. Finally, intraclass correlation analyses were performed (in cases of occasional missing values by using the following settings: single measures; random effects; 95% confidence interval).

## Results

### Patient characteristics

The study population included 90 subjects (38 females, 52 males), all with intravenous thrombolysis before the MT (weight-adapted dosage of 69.2 ± 13.2 mg [mean ± standard deviation] of alteplase 1 mg/mL; Table 1), without a history of prior stroke. The mean age was 73.3 ± 13.1 years and the mean body mass index was 27.1 ± 6.3 kg/m². On average, the time from symptom onset until admission was 136.2 ± 84.5 min, with an NIHSS of 16 (interquartile interval [IQR] 8) upon admission, and most often, the MCA is the occluded vessel (46, 51.1%). During MT, a mean of 1.7 ± 1.0 passages were performed until TICI evaluation (TICI 0, *n* = 4; TICI 1, *n* = 0; TICI 2a, *n* = 3; TICI 2b, *n* = 19; TICI 3, *n* = 44). The official TICI score from the radiological reports was reevaluated by one reader (Cohen’s κ 0.765, 95% confidence interval 0.66–0.87, *p* < 0.001). After 24 h, the median NIHSS decreased to 12 (IQR 12), and upon discharge to 4 (IQR 11).

**Table 1:** Study population characteristics

			Subgroup	Mean/median/number	%	Missing
Age				73.3 ± 13.1		0
Sex			Female/all	38/90	42.2	0
Body mass index				27.1 ± 6.3		23
Baseline medication	Acetylsalicylic acid		Yes	30	35.3	5
	P2Y12 inhibitor		Yes	0	0	5
	Vitamin K antagonists		Yes	1	1.1	5
	Angiotensin—converting enzyme inhibitors		Yes	24	26.7	35
	Beta-blocker		Yes	24	26.7	35
	Loop diuretics		Yes	8	8.9	35
	Statins		Yes	14	15.6	35
Cardiovascular risk factors		Arterial hypertension	Yes	50	70.4	0
		Diabetes mellitus	Yes	12	16.9	2
		Dyslipidemia	Yes	31	43.7	4
		Atrial fibrillation	Yes	21	29.6	3
		Smoking status	Yes	6	8.5	8
			Previous	17	23.9	
			No	59	65.6	
Premorbidity		Heart failure	Yes	3	4.2	11
		Coronary artery disease	Yes	13	18.3	11
		Prior transitory ischaemic attack	Yes	2	2.8	11
		Cancer	Yes	6	8.5	11
*T*_adm_: time from symptom onset to admission, [min]				131.3 ± 82.3		30
*T*_postMT_: time from symptom onset to flow restoration, [min]				237.7 ± 9 3.1		39
Intravenous thrombolysis received				90		0
Intravenous thrombolysis doses, [mg]				69.2 ± 13.2		0
Treated territory			ACA	28	31.1	0
			MCA	43	47.8	
			BA/PCA	19	21.1	
Number of passages				1.65 ± 0.95		5
Adverse events (during MT)		Device malfunction	Yes	1	8.3	0
		Vasospasm	Yes	2	16.7	
		Clot migration embolus	Yes	1	8.3	
		Dissection	Yes	2	16.7	
		Further Stroke	Yes	1	8.3	
		Intracranial haemorrhage	Yes	0	0.0	
		Others	Yes	5	41.7	
TICI score			0	6	5.1	0
			1	0	0.0	
			2a	3	2.6	
			2b	27	23.1	
			3	54	46.2	
NIHSS		On admission		16 (IQR 8)		0
		24 h after MT		12 (IQR 12)		0
		Discharge		4 (IQR 11)		0

*ACA* Anterior cerebral artery, *BA/PCA* Basilar artery occlusion with hypoplastic posterior communicating artery, *MCA* Middle cerebral artery, *NIHSS* National Institutes of Health Stroke Scale, *TICI* Thrombolysis in cerebral infarction

### Mean perfusion parameter values before and after reperfusion

In each major vessel territory, five ROIs were set into the brain parenchyma on lateral and anterior–posterior views. The determined values (MS, TTP, and CA_max_) were compared between the territory-specific ROI for potential significant differences that could arise from variations in measurements by different readers. High intraclass correlations were observed among the ROIs for all three territories (ACA, MCA, BA/PCA) in separate analyses for the perfusion situation before and after MT (ICC ≥ 0.822; see Supplementary Items S1a–d).

Intraclass correlation analyses revealed no significant differences between these five ROIs in 9 out of 12 measurements in the ACA territory, in 12 out of 12 measurements in the MCA territory, and in 12 out of 12 measurements in the BA/PCA territory (ICC > 0.8; *p* < 0.001; Supplemental Items S1a–d). As a result, the values of the five ROIs (anterior–posterior/lateral) for each parameter were subsequently aggregated as the mean.

The mean values of the five ROI of: (i) CA maximum (CA_max_); (ii) the steepest increase of the CA curve (MS); and (iii) time to CA peak (TTP) were analysed. The values of CA_max_ significantly increased after MT, equally to MS (*p* < 0.001 both globally and for the three territories; see Table 2). Referring to the entire brain parenchyma (global), CA_max_ increased from 0.04 ± 0.03 a.u. before mechanical reperfusion to 0.21 ± 0.09 a.u. after the completion of interventional therapy (*p* < 0.001; Table 2). The values of MS increased from 0.03 ± 0.02 (mean ± standard deviation) a.u./s to 0.11 ± 0.05 a.u./s after MT (*p* < 0.001; Table 2). The TTP significantly decreased in the MCA territory compared to imaging before MT (3.2 ± 1.3 s *versus* 2.5 ± 1.1 s, *p* = 0.018).

**Table 2:** Comparing the DSA perfusion parameters stratified according to the initially hypoperfused major brain vessel territories of ACA (*n* = 28), MCA (*n* = 43), and PCA (*n* = 19), as well as stratified by the postinterventional TICI scores

	Mean values before (pre) and after (post) intervention								
Territory	CA_max_, [pre]	CA_max_, [post]	*p*-value	MS, [pre]	MS, [post]	*p*-value	TTP, [pre]	TTP, [post]	*p*-value
Global	0.04 ± 0.03	0.21 ± 0.09	< 0.001	0.03 ± 0.02	0.11 ± 0.05	< 0.001	2.67 ± 1.15	2.53 ± 0.98	0.64
ACA	0.03 ± 0.02	0.2 ± 0.13	< 0.001	0.02 ± 0.01	0.1 ± 0.06	< 0.001	2.3 ± 0.6	2.6 ± 0.75	0.18
MCA	0.05 ± 0.03	0.21 ± 0.08	< 0.001	0.03 ± 0.02	0.11 ± 0.06	< 0.001	3.19 ± 1.31	2.5 ± 1.1	0.018
PCA	0.04 ± 0.03	0.21 ± 0.08	< 0.001	0.03 ± 0.02	0.12 ± 0.05	< 0.001	2.01 ± 0.92	2.24 ± 0.82	0.49

	Differences (Δ) in the parameters before and after intervention in comparison of the radiation beam path anterior–posterior (ap) *versus* lateral (lat)								
Territory	ΔCA_max_, [ap]	ΔCA_max_, [lat]	*p*-value	ΔMS, [ap]	ΔMS, [lat]	*p*-value	ΔTTP, [ap]	ΔTTP, [lat]	*p*-value
Global	0.22 ± 0.16	0.12 ± 0.06	< 0.001	0.11 ± 0.09	0.06 ± 0.05	< 0.001	- 0.27 ± 1.92	- 0.01 ± 1.52	0.028
ACA	0.24 ± 0.24	0.11 ± 0.04	0.034	0.11 ± 0.10	0.06 ± 0.04	0.034	0.41 ± 1.53	0.29 ± 0.59	0.128
MCA	0.22 ± 0.12	0.10 ± 0.06	< 0.001	0.12 ± 0.08	0.05 ± 0.05	< 0.001	- 0.93 ± 2.11	- 0.44 ± 1.84	0.192
PCA	0.17 ± 0.1	0.17 ± 0.07	0.49	0.08 ± 0.05	0.09 ± 0.06	0.49	- 0.15 ± 1.37	0.6 ± 1.33	0.013

The difference between the baseline and the CA_max_ was defined as a.u., the TTP in seconds (s), and the MS of the CA time concentration curve (MS) as a.u./s

*ACA* Anterior cerebral artery, *MCA* Middle cerebral artery, *PCA* Posterior cerebral artery, *CA*_*max*_ Difference between the baseline and the maximum of the CA concentration, *MS* Maximum slope of the C(t)-curve, *TICI* Thrombolysis in cerebral infarction, *TTP* Time to peak

When comparing the beam paths (anterior–posterior *versus* lateral), multiple significant differences were observed in the differences of perfusion parameters (Table 2, lower section). For example, there was a notable difference in CA_max_ between the anterior–posterior to the lateral beam path (0.22 ± 0.12 *versus* 0.1 ± 0.06; see Table 2). The significant differences observed in the parameters based on the beam path induced the conductance of principal component analyses to identify potential patterns, particularly associated with the direction of the beam path (anterior–posterior/lateral).

**Fig. 3:**
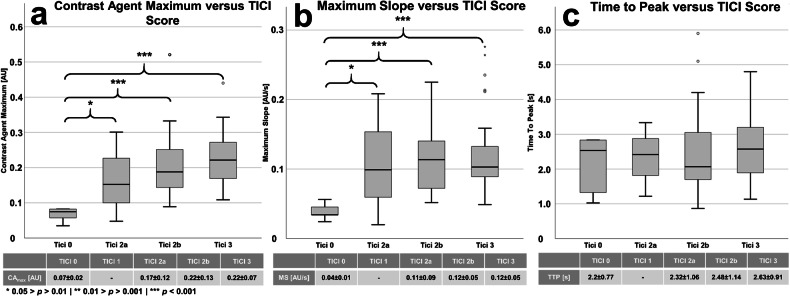
Stratification of DSA-based perfusion parameters according to the classification according to the TICI score. Significant differences were observed in the parameters (**a**) CA_max_ and (**b**) MS (*p = 0.05–0.01; **p = 0.01–0.001; ***p < 0.001), unlike in the (**c**) TTP

Following the stratification of perfusion metrics determined during the final assessment, significant differences were observed between the classes of the TICI score only in the parameters CA_max_ and MS. In particular, significant differences in CA_max_ and MS were evident between the classes TICI 2a, 2b, and 3 on one hand, and TICI 0 on the other hand (Fig. 3). In the case of TICI 0, both CA_max_ (Fig. 3a) and MS (Fig. 3b) were significantly lower compared to, for example, TICI 3 (0.07 ± 0.02 a.u. and 0.04 ± 0.01 a.u./s *versus*. 0.22 ± 0.07 a.u. and 0.12 ± 0.05 a.u./s). Regarding the TTP, no significant differences were observed (Fig. 3c).

### Pattern detection for multiple regression analyses

A principal component analysis was performed to extract the most important independent factors and to identify possible variables for regression models (Supplementary Items S2a, b). Kaiser–Meye–Olkin measure of sampling adequacy was 0.503 and Bartlett’s test of sphericity (*p* < 0.001) with factor eigenvalues ≥ 1 (which basically means that the quality of analysed data was sufficient and usable for factor analysis [33, 34]).

The items “mRS before admission”, “NIHSS_24h_” and “NIHSS_dc_” had the highest loadings on the factor models (along the factor solutions each ≥ 4 times with values ≥ 0.2). The mean values of CA_max_ and MS after MT loaded the most in the third-factor solution (0.919 and 0.946), whereas the TTP loaded the most in the second-factor solution (-0.816). Regardless of the values measured in the anterior–posterior or lateral radiation beam paths, strong loadings were observable.

Subjects’ age, modified Rankin scale (mRS) before admission, NIHSS_adm_, the time between symptom onset/time of symptom recognition until flow restoration (*T*_flow_), and each mean perfusion parameter after MT (CA_max_, MS, TTP) were selected for regression analyses, representing a comparable informational basis to the evaluation of the TICI score (see Supplementary Item S2b).

### Prediction of neurological deficits following MT

Multiple linear regression analysis with backward elimination and stepwise variable selection included those parameters for determination of regression models to estimate the NIHSS_24h_ and NIHSS_dc_, which have been identified by the principal component analyses: subjects’ age, mRS before admission, the *T*_flow_, and the NIHSS_adm_. On the one hand, those parameters were combined with perfusion parameters after MT in one calculation model, and on the other hand, they were combined with the TICI score. Backward elimination and stepwise variable selection gradually eliminated parameters in the models with no significant influence on the equations.

**Fig. 4:**
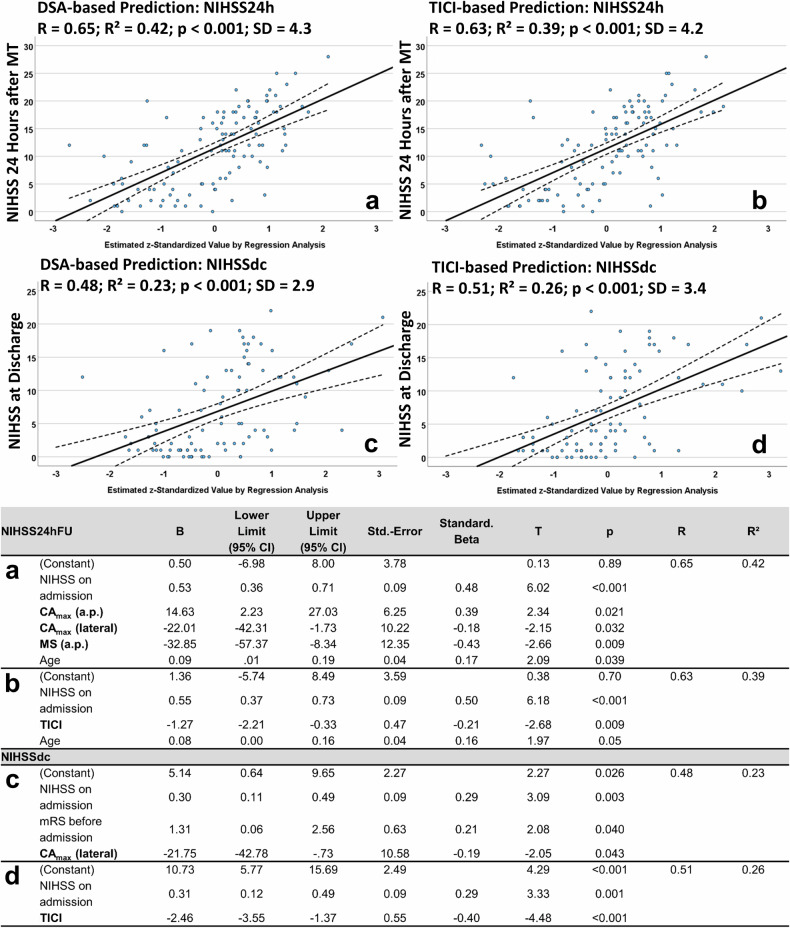
Results of the multiple linear regression analysis with backward elimination and stepwise variable selection to predict the NIHSS score. Scatter plots of multiple linear regression analyses with backward elimination and stepwise variable selection included: (i) the NIHSS score on admission; (ii) age; (iii) preexisting mRS due to other comorbidities; and (iv) perfusions parameters or the TICI score to estimate the NIHSS scores after 24 h (**a**, **b**) and at discharge (**c**, **d**). Perfusion parameters included the mean values of the regions of interest obtained in both anterior–posterior (ap) and lateral projections, calculated after MT (“post”). The values of the regression equations were standardised (*z*-standardised) and plotted against their respective predicted variables (NIHSS_24h_ or NIHSS_dc_) on a coordinate system. Consequently, the values should be understood in such a way that a value of 1 corresponds to the regression equation value calculated as the first standard deviation. A value of -2 thus corresponds to values that are two standard deviations below the mean. Here, the linear terms for the utilised regression models are visualised. **a**, **c** Represent the regression equations using perfusion parameters from DSA, while panels **b**, **d** represent the regression equations using the TICI score. The central bold line corresponds to the mean of the equation, while the dashed line represents the upper and lower limits of the 95% confidence interval (CI), respectively. *R*, *R*², and the standard deviation of the entire regression equation were reported

**Fig. 5:**
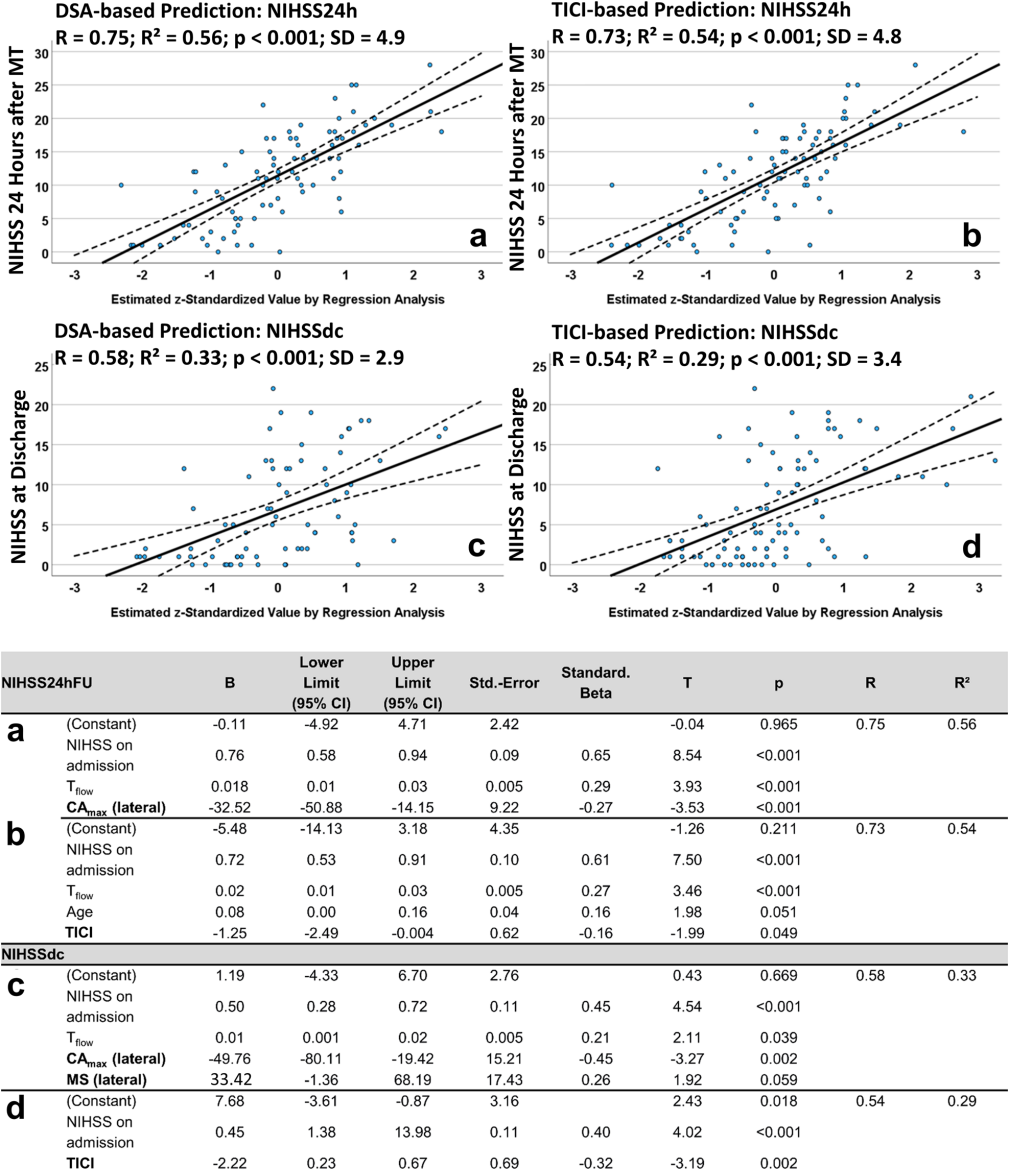
Results of the multiple linear regression analysis with backward elimination and stepwise variable selection to predict the NIHSS by the additional use of *T*_flow_. Scatter plots of multiple linear regression analyses with backward elimination and stepwise variable selection included the time interval from symptom onset or last seen well to flow restoration (*T*_flow_), NIHSS on admission, age, and preexisting mRS due to other comorbidities to estimate the NIHSS scores after 24 h (NIHSS_24h_, upper both images) and at discharge (NIHSS_dc_, lower both images). Perfusion parameters included the mean values of the regions of interest obtained in both anterior–posterior (ap) and lateral projections, calculated after MT (“post”). The values of the regression equations were standardised (*z*-standardised) and plotted against their respective predicted variables (NIHSS_24h_ or NIHSS_dc_) on a coordinate system. Consequently, the values should be understood in such a way that a value of 1 corresponds to the regression equation value calculated as the first standard deviation. A value of -2 thus corresponds to values that are two standard deviations below the mean. Here, the linear terms for the utilised regression models are visualised. **a**, **c** Represent the regression equations using perfusion parameters from DSA, while panels **b**, **d** represent the regression equations using the TICI score. The central bold line corresponds to the mean of the equation, while the dashed line represents the upper and lower limits of the 95% confidence interval (CI), respectively. *R*, *R*², and the standard deviation of the entire regression equation (SD) were reported

The analyses revealed that using the NIHSS_adm_, age, and a preexisting mRS due to other comorbidities, the NIHSS_24h_ and NIHSS_dc_ were roughly comparable when determined by the TICI score and perfusion parameters (Δ*R*² = 0.03 each; Eqs. 3 and 5; see Fig. 4). Adding the *T*_flow_ to the regression models revealed no significant superiority of using the TICI score or perfusion parameters (Δ*R*² = 0.02 and 0.04; Eqs. 4 and 6; see Fig. 5). Whereas the predictive power of this model using the *T*_flow_ was superior to the previous one (*R* = 0.73–0.75 *versus* *R* = 0.63–0.65; *p* < 0.001 each; Figs. 4 and 5). The regression analyses were described by the following equations (including DSA-based perfusion parameters; Eqs. 3−6):

Equation 3 (see Fig. 4a)

3 $${{\rm{NIHSS}}}_{{\rm{24h}}}= 	 0.5+0.53* {{\rm{NIHSS}}}_{{\rm{adm}}}+14.63* {{\rm{CA}}}_{\max }\left(a.p.\right) \\ 	 -22.01* {{\rm{CA}}}_{\max }({{\rm{lateral}}})-32.85* {{\rm{MS}}}({{\rm{anterior}}}-{{\rm{posterior}}})\\ 	 +0.09* {{\rm{Age}}}$$

Equation 4 (see Fig. 5a; with *T*_flow_)

4 $${{\rm{NIHSS}}}_{{\rm{24h}}} = 	 -0.11+0.76* {{\rm{NIHSS}}}_{{\rm{adm}}}\,+0.018* {T}_{{\rm{flow}}} \\ 	 -32.52* {{\rm{CA}}}_{\max }\,({{\rm{lateral}}})$$

Equation 5 (see Fig. 4c)

5 $${{\rm{NIHSS}}}_{{\rm{dc}}}= 	 5.14+0.3* {{\rm{NIHSS}}}_{{\rm{adm}}}+1.31* {{\rm{mRS}}}\left({{\rm{preexisting}}}\right) \\ 	 -21.75* {{\rm{CA}}}_{\max }({{\rm{lateral}}})$$

Equation 6 (see Fig. 5c; with *T*_flow_)

6 $${{\rm{NIHSS}}}_{{\rm{dc}}}= 	 1.19+0.5* {{\rm{NIHSS}}}_{{\rm{adm}}}+0.01* {T}_{{\rm{flow}}} \\ 	 -49.76* {{\rm{CA}}}_{\max }\left({{\rm{lateral}}}\right)+33.42* {{\rm{MS}}}({{\rm{lateral}}})$$

### Congruence analysis

**Fig. 6:**
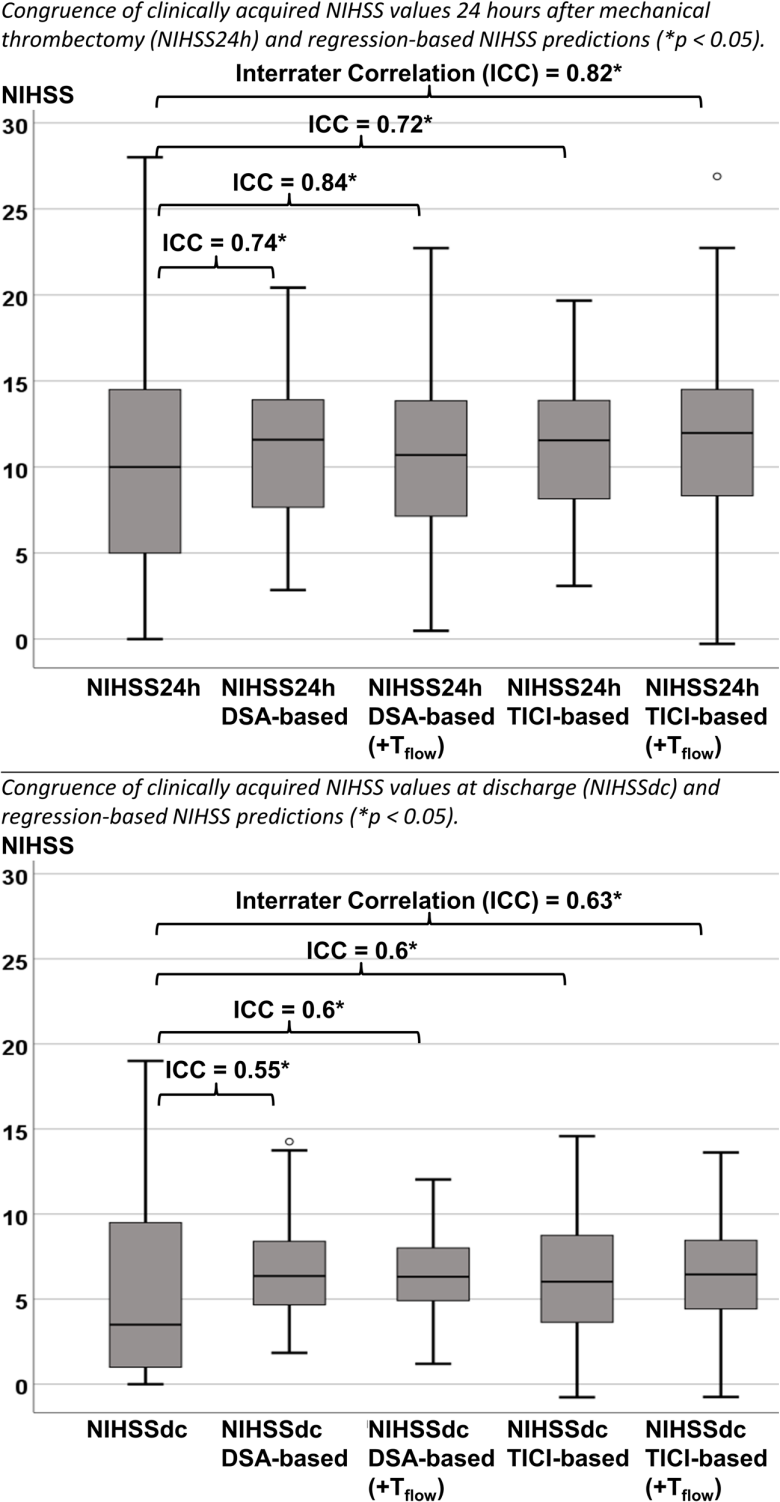
Congruence of clinically acquired NIHSS values with predicted NIHSS (regression-based)

The NIHSS_24h_ and NIHSS_dc_ were calculated using the regression equations extracted from Figs. 4 and 5, and are presented in Supplemental Item S3 compared to the actual NIHSS values. Intraclass correlation analyses revealed an ICC between 0.55 and 0.84 for the DSA-based NIHSS values compared to the actual NIHSS values, and an ICC between 0.6 and 0.82 for the TICI-based equations (*p* < 0.001; Fig. 6).

## Discussion

**Fig. 7:**
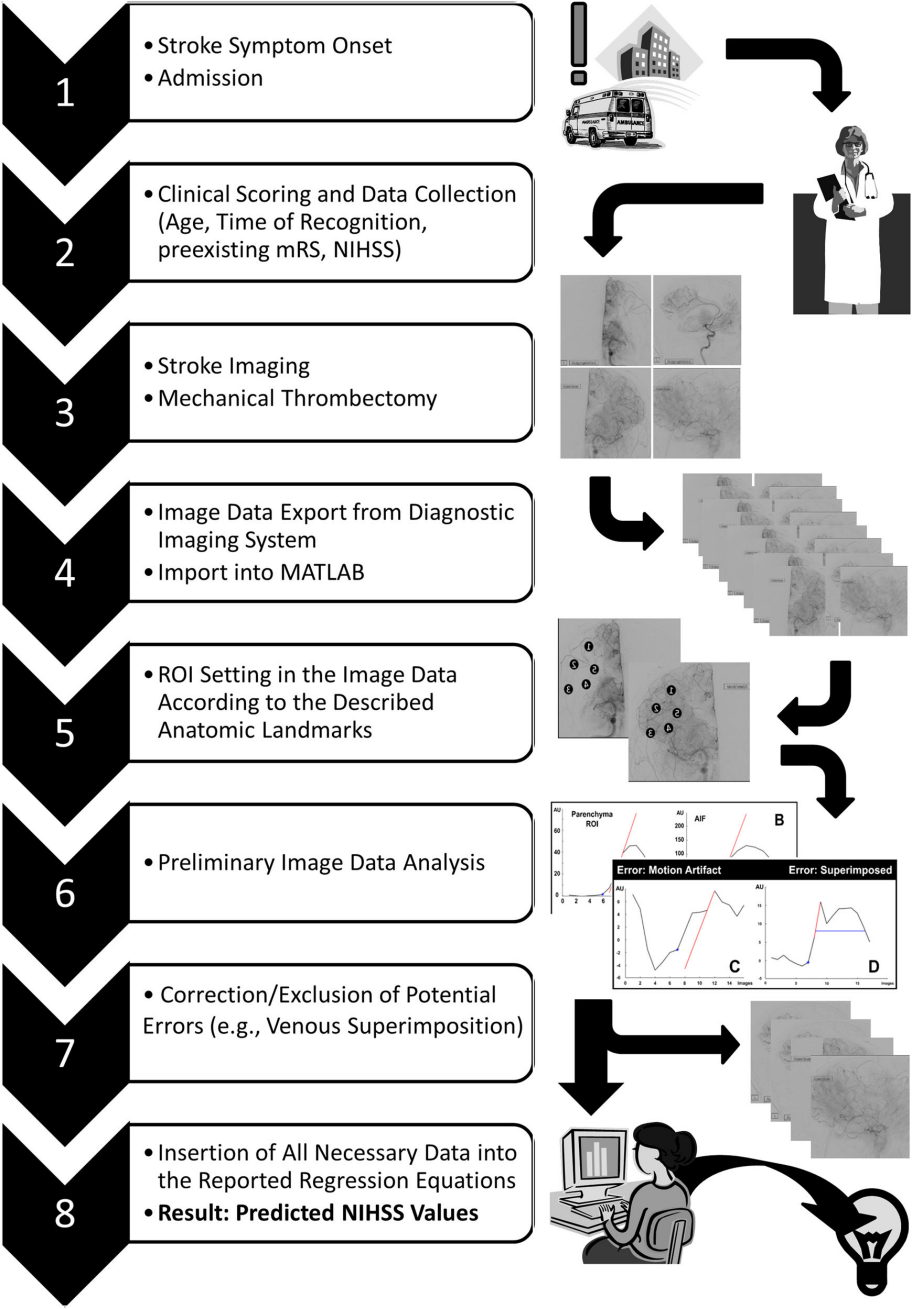
Process of data acquisition and evaluation

In this study, the measurement of cerebral perfusion by DSA was performed using five ROIs, each placed within the major vascular territories. There were no relevant differences between the five ROIs within the ap or lateral radiation beam path. However, significant differences in the perfusion parameters before and after MT were observed across both beam angles (anterior–posterior/lateral): in anterior–posterior, the values were higher than in the lateral angulation. After MT, the mean values of CA_max_ and MS increased and the TTP decreased significantly. The following factors were identified as relevant for predicting the NIHSS: CA_max_ and MS after MT, the patient’s age, mRS before admission, NIHSS_adm_, the time from symptom onset or recognition until flow restoration, and each mean parameter after MT. The TICI score and perfusion parameters were equivalent in predicting the predefined auxiliary parameters (NIHSS_24h_ and NIHSS_dc_). The variances of the reevaluated TICI scores (Cohen κ) were within the ranges reported in the literature (κ 0.36–0.82) [14]. The ICCs for the extrapolated NIHSS_24h_ were comparable to those reported by other authors, and for the evaluation at discharge they were lower (Fig. 6) [27, 35]. Figure 7 provides a complete visualisation of the data collection and analysis process.

In our analyses, the NIHSS_adm_ had a significant role in the phase of (sub-)acute treatment to predict the NIHSS_24h_, as indicated in previous studies, however, the influence of NIHSS_adm_ on the NIHSS_dc_ was lower [36–38]. This could be interpreted as the diminishing influence of acute therapy in the longer period of post-acute treatment. Several predicting models in previous studies were able to obtain similar results [36–38]. In addition, the longer acute phase care takes (and the more complex the endovascular therapy), the worse the expected outcome. This is in line with observations in other studies [36, 39, 40].

After stratification into the TICI score classes, interclass differences were observed in CA_max_ and MS. These differences were particularly significant between TICI 0 and the higher classes (such as TICI 2a or TICI 3). Particularly concerning MS, there were some outliers observed in the TICI 3 class that could potentially upwardly adjust the upper maximum of the measurements. Overall, it is presumed that with a larger sample size and the resulting subdivision into major vascular territories, there might be significant differences in the measurements between the higher TICI classifications. Furthermore, at the current moment, it is not entirely clear which specific changes are being captured by the perfusion measurements, especially considering the currently non-significant results of TTP after stratification into TICI classes. Taking into account other models or subdivisions, including clinical outcomes, could potentially provide an unforeseen benefit of perfusion over the semi-quantitative, expert-based reperfusion outcome classification. Taking into consideration the latest review articles, it has been reported that there is a general consensus that post-recanalisation perfusion deficits would lead to an unfavourable outcome, particularly concerning persistently hypoperfused brain regions [15, 16]. It has been noted that postinterventional hyperperfusions, in particular, have been observed multiple times and appear to be associated with both unfavourable and favourable outcomes [15, 16, 41]. Therefore, the correlation of brain perfusion should include more clinical parameters to reflect the role of post-interventional perfusion conditions [15, 41]. For instance, the so-called hyperperfusion syndrome is known, which can accompany intracranial haemorrhages after the reopening of occluded vessels [16, 42, 43].

So far, there are few comparable studies. van der Sluijs et al [17] examined the ratio of reperfused to nonreperfused pixels in the brain parenchyma and were able to achieve similar classifications as expert-assessed reperfusion outcomes in proximal occlusions, as is also the case in the present study. The authors were able to more accurately determine functional outcomes by incorporating the so-called “autoTICI”, compared to predictions based solely on patient data. A comparability limitation is that the autoTICI was applied only to M1, M2, and internal carotid artery occlusions, and not to proximal occlusions within all great vessel territories, as was conducted in the present study [17].

Approaches that specifically considered the perfusion status of the precentral cortex (so-called “eloquence-based reperfusion”) were conventionally evaluated by experts regarding reperfusion success. Raychev et al [19] utilised a three-tiered system (no flow, partial flow, complete reperfusion) for multivariate analyses, applied to a dataset primarily consisting of confirmed TICI 2a and 2b cases. This eloquence-based approach, however, demonstrated superiority over the volume-based approach of the TICI score [19]. Pressman et al [18] also examined the reperfusion status in brain regions with a clear impact on eloquence using a three-tier grading system and correlated these findings with NIHSS and mRS scores. These studies investigating eloquence-based approaches demonstrated that accounting for eloquent regions (*e.g*., the precentral cortex) has a significant impact on clinical outcomes, as measured by NIHSS and mRS scores [18, 19].

The AFTERMATH study [20] also examined DSA-based and expert-based assessments of reperfusion success in 26 patients, stratifying them into four subgroups to specifically compare discrepant cases (hypoperfusion in DSA *versus* complete reperfusion assessed by experts with *n* = 2 subjects; complete reperfusion in DSA *versus* hypoperfusion assessed by experts with *n* = 3 subjects). The evaluation of the DSA images was conducted similarly to the present study, by analysing the CA bolus and the contrast enhancement of the brain parenchyma over time [20]. The authors did not find a correlation with clinical parameters [20]. However, they were able to identify false positive TICI 3 cases based on expert assessments using DSA-based evaluations [20].

Nielsen et al [14] aimed to extract a TICI score that incorporated the following dimensions: (i) time-based changes in pixel alterations in DSA; (ii) eloquent brain regions; and (iii) known risk factors that could affect the assessment of the TICI score. This study included 236 patients with M1 occlusions and achieved a valid agreement with the gold standard of expert-based readings (Cohen κ 0.61) [14]. The methodology was subsequently reevaluated using 95 external datasets, yielding a κ of 0.52 [14]. Nevertheless, these agreements were comparable with the known interobserver variabilities for TICI scoring.

In future studies, the significance within the large vessel territories should be tested in larger patient cohorts and, if necessary, in combination with clinical parameters with less susceptibility to interference, such as the NIHSS used in the present study. Furthermore, a validation with an external cohort would underpin the method’s reliability. Additionally, it should be considered whether excluding cortical veins and arteries is required for perfusion measurement after AIS (unlike, for example, in delayed cerebral ischaemia). Nonetheless, the current method would allow for semiautomated perfusion assessment, with trained personnel defining the ROIs and subsequent automated analysis. Approaches for the automatic segmentation of arteries, veins, and capillaries have already been investigated in a study with promising results. Although the presented method is technically simpler to implement than other comparable methods, advanced programming knowledge is required. Furthermore, an extended protocol (up to the completion of the venous phase) would better represent the outflow, thereby enabling the calculation of AUC and FWHM. Ultimately, special attention should be given to regions involved in the development of neurological deficits (areas of eloquence), such as the precentral cortex, occipital lobe, pons, and cerebellum. Future studies should aim to develop an objective tool for expert-independent multicentre studies.

There are several limitations to our study. First, the majority of cases in our dataset (47%) were AIS in the MCA territory. The small sample size consisting primarily of proximal occlusions may limit the generalizability of the results, and additional confirmation with larger cohorts is necessary. Furthermore, all patients received intravenous thrombolysis and, in combination with MT, predominantly achieved a high rate of revascularisation, resulting in a low number of TICI 0 and 1 results (strong limitation). Pooling a small number of TICI 0 cases with a relatively high number of TICI 2a/b and 3 cases could introduce bias, particularly with regard to the reported means of perfusion parameters when stratified by TICI score. On the other hand, the TICI scores recorded in the findings were used for analyses, conducting neurointerventionalists themselves had evaluated, potentially contributing to an inter-observer bias. Despite the resistance to interrater variabilities in assessing reperfusion success after MT of proximal occlusions with semiquantitative methods [12] and the challenging differentiation between TICI score 2b and 3 [8], neurological deficits were equally predictable using either the TICI score or the new semiautomated method.

From a technical perspective, there were also some observations to criticise. While the CA curves derived from the DSA data were examined for potential sources of error (*e.g*., superimposed venous filling), similar effects masked by the parenchymal phase C(t)-curves could not be ruled out and could have an impact on our results. We addressed venous superimposed or motion-distorted data by exclusion and repeated measurements at immediately adjacent locations. Additionally, C(t)-curves were rarely acquired until the completion of the venous contrast phase in most cases due to radiation protection reasons. Therefore, the AUC and FWHM could not be examined within our analyses. A premature venous filling, typically associated with a poorer outcome, could be evaluated more accurately with an optimised protocol (approximately 25% increase in radiation dose when waiting for venous outflow). From the current point of knowledge, a higher image acquisition rate than two images/s does not appear necessary for reperfusion evaluation after AIS, as the CA curves could be reliably captured. Furthermore, there was only one reader who standardised five ROI in the brain parenchyma. The impact of the lack of inter-reader reliability was investigated by examining whether there were significant differences between the different ROIs within a territory. In this regard, intraclass correlations were conducted, predominantly revealing that no relevant differences were observed in the ROI within the same territory. This suggests that placing an ROI at any location in the initially hypoperfused brain parenchyma would yield nearly identical results. Nevertheless, this point must be considered a major limitation.

Ultimately, to validate our results, our methodology should be tested with an external cohort. To conclude, DSA-based brain perfusion measurements pre- and post-MT for AISs reliably predict reduced neurological deficits in patients with proximal occlusion patterns and with intravenous thrombolysis received. Subsequently, this technology could reduce the need for expertise in DSA image evaluation, facilitating enhanced data comparison across neurointerventionalists and medical centres. Overall, it highlights the reliability of DSA-based methods for brain perfusion measurement, particularly in potential future automated assessments.

## Supplementary information

**Additional file 1:** **Supplementary Item 1a:** The intraclass correlations (ICC) were calculated for each of the 5 ROIs per parameter in anteroposterior projection before mechanical thrombectomy. Row A) represents the contrast agent maximum (CA_max_), B) depicts the maximum slope of the incoming contrast agent (MS), and C) illustrates the time to contrast agent maximum (TTP). Subpoints 1-3 encode for the territory of the anterior cerebral artery (1), the middle cerebral artery (2), and the posterior cerebral artery (3). **Supplementary Item 1b:** The intraclass correlations (ICC) were calculated for each of the 5 ROIs per parameter in lateral projection before mechanical thrombectomy. Row A) represents the contrast agent maximum (CA_max_), B) depicts the maximum slope of the incoming contrast agent (MS), and C) illustrates the time to contrast agent maximum (TTP). Subpoints 1-3 encode for the territory of the anterior cerebral artery (A1-C1), the middle cerebral artery (A2-C2), and the posterior cerebral artery (A3-C3). **Supplementary Item 1c:** The intraclass correlations (ICC) were calculated for each of the 5 ROIs per parameter in anteroposterior projection after mechanical thrombectomy. Row A) represents the contrast agent maximum (CA_max_), B) depicts the maximum slope of the incoming contrast agent (MS), and C) illustrates the time to contrast agent maximum (TTP). Subpoints 1-3 encode for the territory of the anterior cerebral artery (A1-C1), the middle cerebral artery (A2-C2), and the posterior cerebral artery (A3-C3). **Supplementary Item 1d:** The intraclass correlations (ICC) were calculated for each of the 5 ROIs per parameter in lateral projection after mechanical thrombectomy. Row A) represents the contrast agent maximum (CA_max_), B) depicts the maximum slope of the incoming contrast agent (MS), and C) illustrates the time to contrast agent maximum (TTP). Subpoints 1-3 encode for the territory of the anterior cerebral artery (A1-C1), the middle cerebral artery (A2-C2), and the posterior cerebral artery (A3-C3). **Supplemental Item 2a:** Principal Component Analysis (KMO- and Barlett-Test, and Variance analysis). **Supplemental Item 2b:** Principal Component Analysis (Rotated component matrix). **Supplemental Item 3:** Comparison of Real NIHSS Values and Regression-Based NIHSS Predictions Post-Thrombectomy.

## Data Availability

The study data are available from the corresponding author upon reasonable request from any qualified investigator. The request should be based on a scientific hypothesis and be approved by a (local) ethical committee. Any request must be made in writing. Data will be saved for ten years after publishing (according to GCP guidelines). The MATLAB code for data export and analysis is available upon reasonable request and on a collaborative basis. Additionally, inquiries regarding programming or more technical aspects can be directed to AK. The core functions’ codes have been uploaded by AK to the MATLAB file exchange platform (https://de.mathworks.com/matlabcentral/fileexchange/172029-functions-for-first-pass-perfusion-evaluation).

## Abbreviations

a.u: Arbitrary units
A1: First segment of the anterior cerebral artery
ACA: Anterior cerebral artery
AIF: Arterial input function
AIS: Acute ischaemic stroke
AUC: Area under the curve
BA: Basilar artery
C(t)-curve: Function describing time dependent changes in contrasting the brain parenchyma
CA: Contrast agent
CA_max_: Difference between the baseline and the maximum of the CA concentration
DSA: Digital subtraction angiography
FWHM: Full width of half maximum
ICC: Intraclass correlation coefficient
IQR: Interquartile interval
M1: First segment of the middle cerebral artery
MCA: Middle cerebral artery
mRS: Modified Rankin scale
MS: Maximum slope of the C(t)-curve
MT: Mechanical thrombectomy
NIHSS: National Institutes of Health Stroke Scale
NIHSS_24h_: NIHSS after 24 h
NIHSS_adm_: NIHSS at admission
NIHSS_dc_: NIHSS at discharge
P1: First segment of the posterior cerebral artery
PCA: Posterior cerebral artery
ROI: Region of interest
*T*_flow_: Time between symptom onset/time of symptom recognition until flow restoration
TICI: Thrombolysis in cerebral infarction
TTP: Time to peak
