# Supplementary material for: DSA-based perfusion parameters versus TICI score after mechanical thrombectomy in acute ischaemic stroke patients: a congruence analysis
Source: Eur Radiol Exp. 2024 Dec 5;8:136. doi: 10.1186/s41747-024-00534-1 (PMC11621293; doi:10.1186/s41747-024-00534-1)
Supplement: Supplementary file 1 — Additional file 1: Supplementary Item 1a: The intraclass correlations (ICC) were calculated for each of the 5 ROIs per parameter in anteroposterior projection before mechanical thrombectomy. Row A) represents the contrast agent maximum (CAmax), B) depicts the maximum slope of the incoming contrast agent (MS), and C) illustrates the time to contrast agent maximum (TTP). Subpoints 1-3 encode for the territory of the anterior cerebral artery (1), the middle cerebral artery (2), and the posterior cerebral artery (3). Supplementary Item 1b: The intraclass correlations (ICC) were calculated for each of the 5 ROIs per parameter in lateral projection before mechanical thrombectomy. Row A) represents the contrast agent maximum (CAmax), B) depicts the maximum slope of the incoming contrast agent (MS), and C) illustrates the time to contrast agent maximum (TTP). Subpoints 1-3 encode for the territory of the anterior cerebral artery (A1-C1), the middle cerebral artery (A2-C2), and the posterior cerebral artery (A3-C3). Supplementary Item 1c: The intraclass correlations (ICC) were calculated for each of the 5 ROIs per parameter in anteroposterior projection after mechanical thrombectomy. Row A) represents the contrast agent maximum (CAmax), B) depicts the maximum slope of the incoming contrast agent (MS), and C) illustrates the time to contrast agent maximum (TTP). Subpoints 1-3 encode for the territory of the anterior cerebral artery (A1-C1), the middle cerebral artery (A2-C2), and the posterior cerebral artery (A3-C3). Supplementary Item 1d: The intraclass correlations (ICC) were calculated for each of the 5 ROIs per parameter in lateral projection after mechanical thrombectomy. Row A) represents the contrast agent maximum (CAmax), B) depicts the maximum slope of the incoming contrast agent (MS), and C) illustrates the time to contrast agent maximum (TTP). Subpoints 1-3 encode for the territory of the anterior cerebral artery (A1-C1), the middle cerebral artery (A2-C2), and th [file 41747_2024_534_MOESM1_ESM.pdf]

DSA-based Perfusion Parameters versus TICI Score after Mechanical Thrombectomy in Acute Ischemic Stroke Patients: A Congruence Analysis

ELECTRONIC SUPPLEMENTARY MATERIAL

Supplementary Item 1a: The intraclass correlations (ICC) were calculated for each of the 5 ROIs per parameter in anteroposterior projection before mechanical thrombectomy. Row A) represents the contrast agent maximum (CA_{max}), B) depicts the maximum slope of the incoming contrast agent (MS), and C) illustrates the time to contrast agent maximum (TTP). Subpoints 1-3 encode for the territory of the anterior cerebral artery (1), the middle cerebral artery (2), and the posterior cerebral artery (3).

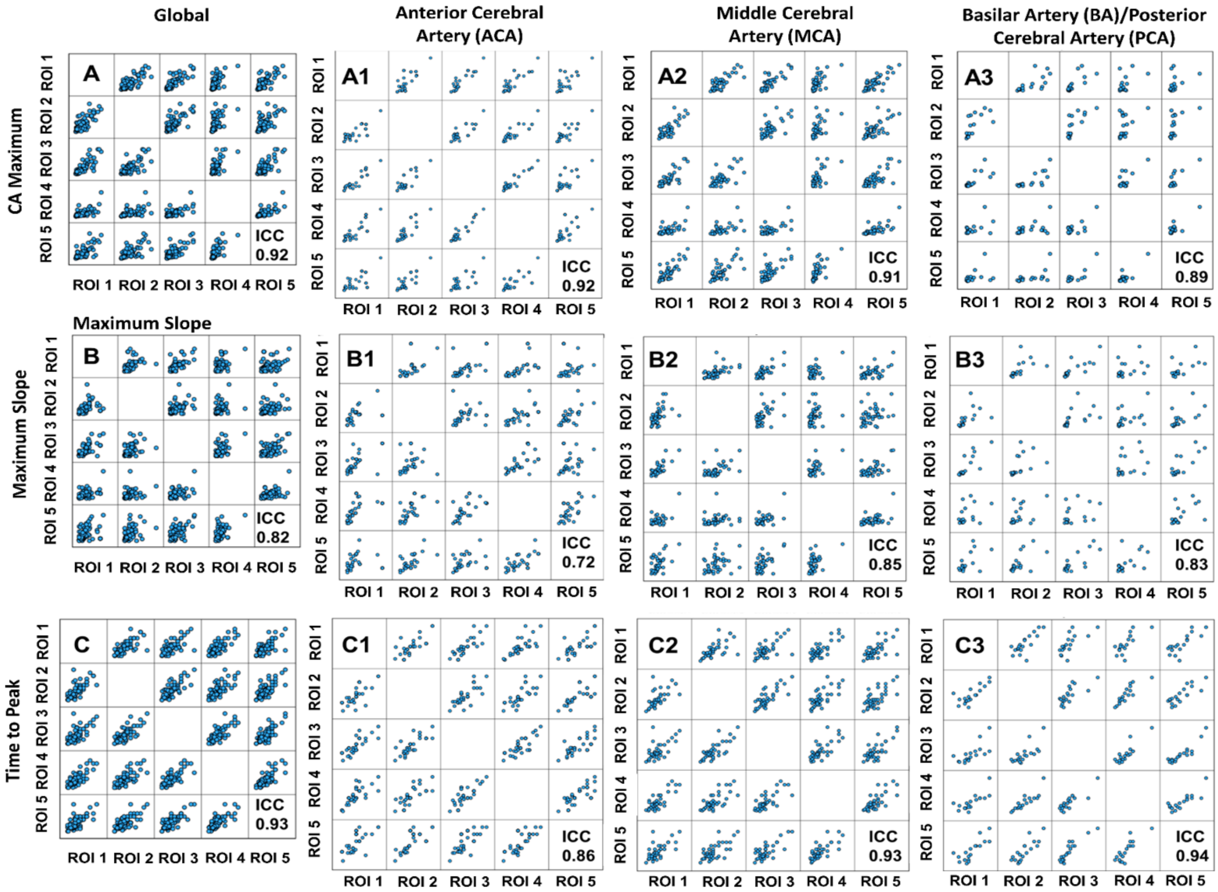

Supplementary Item 1b: The intraclass correlations (ICC) were calculated for each of the 5 ROIs per parameter in lateral projection before mechanical thrombectomy. Row A) represents the contrast agent maximum (CA_{max}), B) depicts the maximum slope of the incoming contrast agent (MS), and C) illustrates the time to contrast agent maximum (TTP). Subpoints 1-3 encode for the territory of the anterior cerebral artery (A1-C1), the middle cerebral artery (A2-C2), and the posterior cerebral artery (A3-C3).

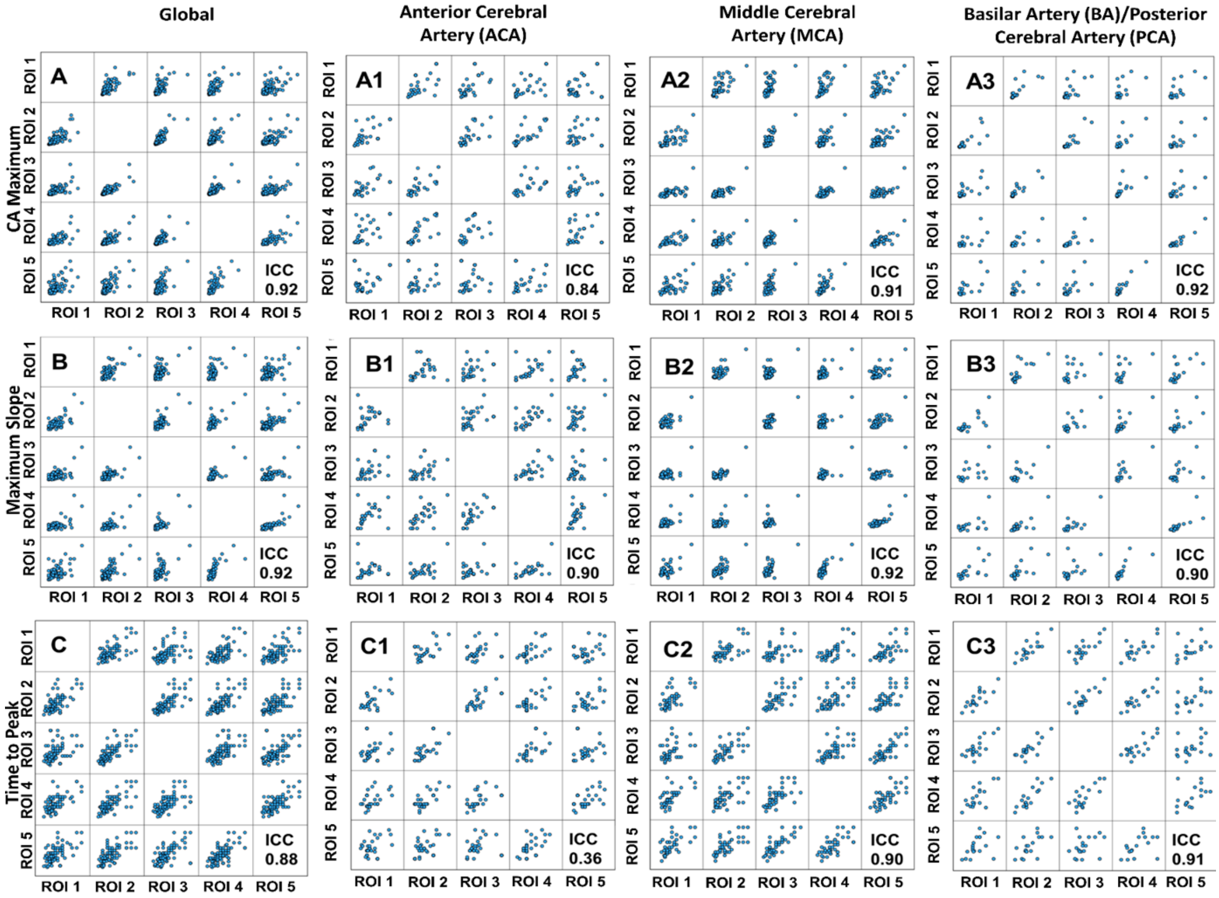

Supplementary Item 1c: The intraclass correlations (ICC) were calculated for each of the 5 ROIs per parameter in anteroposterior projection after mechanical thrombectomy. Row A) represents the contrast agent maximum (CA_{max}), B) depicts the maximum slope of the incoming contrast agent (MS), and C) illustrates the time to contrast agent maximum (TTP). Subpoints 1-3 encode for the territory of the anterior cerebral artery (A1-C1), the middle cerebral artery (A2-C2), and the posterior cerebral artery (A3-C3).

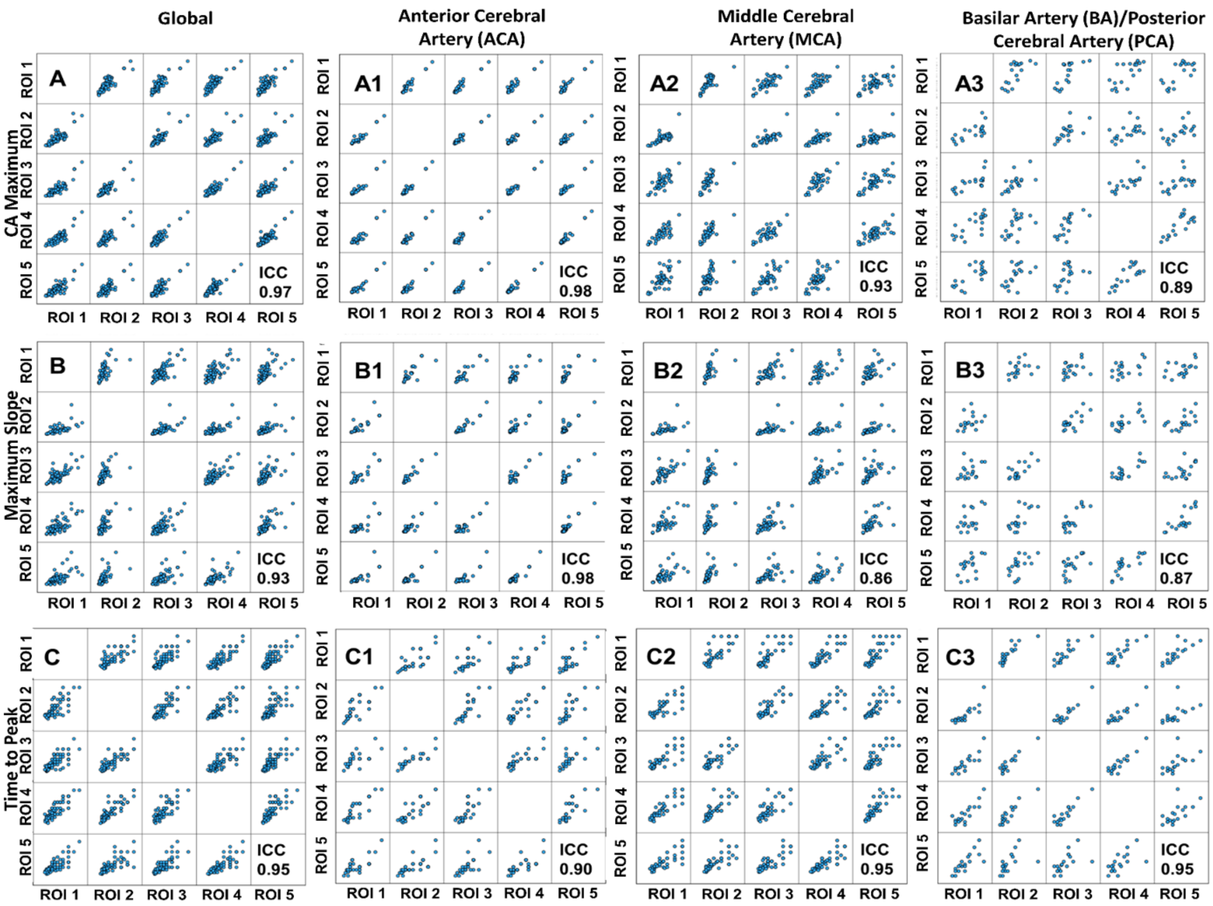

Supplementary Item 1d: The intraclass correlations (ICC) were calculated for each of the 5 ROIs per parameter in lateral projection after mechanical thrombectomy. Row A) represents the contrast agent maximum (CA_{max}), B) depicts the maximum slope of the incoming contrast agent (MS), and C) illustrates the time to contrast agent maximum (TTP). Subpoints 1-3 encode for the territory of the anterior cerebral artery (A1-C1), the middle cerebral artery (A2-C2), and the posterior cerebral artery (A3-C3).

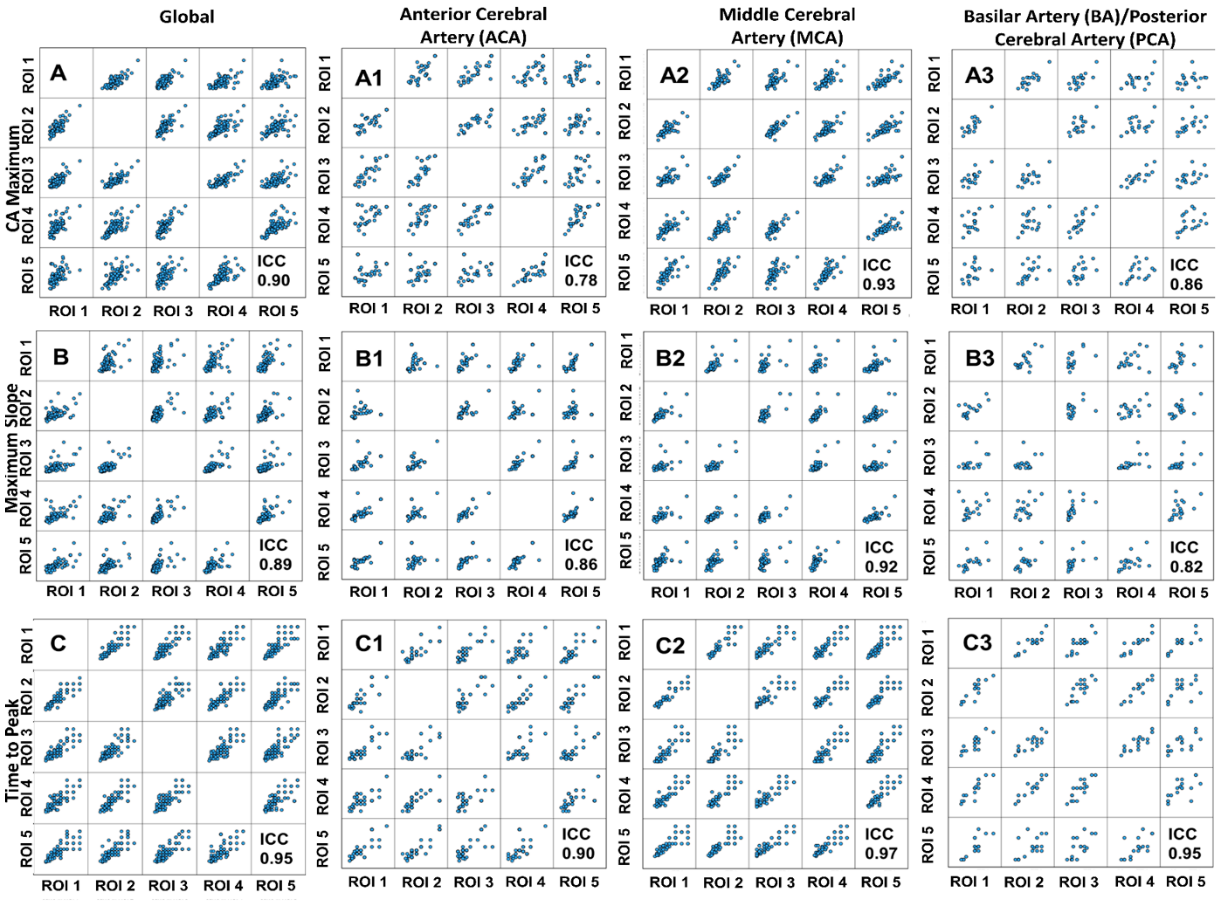

Supplemental Item 2a: Principal Component Analysis (KMO- and Barlett-Test, and Variance analysis).

KMO- and Bartlett-Test		
Measure of Sample Adequacy according to Kaiser-Meyer-Olkin		**0.508**
Bartlett's test of sphericity	Approx. Chi-Squared	275.677
	df	153
	Sig. after Bartlett	**<0.001**

**Explained total variance**									
Component	Initial Eigenvalues			Sums of squared factor loadings for extraction			Rotated sum of squared loadings		
	Overall	% (Variance)	Cumulated %	Overall	% (Variance)	Cumulated %	Overall	% (Variance)	Cumulated %
1	4.452	24.733	24.733	4.452	24.733	24.733	2.743	15.237	15.237
2	2.489	13.830	38.563	2.489	13.830	38.563	2.206	12.254	27.491
3	2.314	12.854	51.417	2.314	12.854	51.417	2.142	11.902	39.393
4	1.727	9.592	61.009	1.727	9.592	61.009	2.114	11.742	51.136
5	1.471	8.170	69.179	1.471	8.170	69.179	1.880	10.445	61.581
6	1.172	6.511	75.690	1.172	6.511	75.690	1.876	10.421	72.002
7	1.073	5.961	81.651	1.073	5.961	81.651	1.737	9.649	**81.651**
8	.695	3.861	85.513						
9	.580	3.221	88.734						
10	.509	2.830	91.564						
11	.438	2.433	93.996						
12	.297	1.651	95.647						
13	.252	1.400	97.047						
14	.180	.998	98.045						
15	.154	.856	98.901						
16	.090	.502	99.403						
17	.056	.310	99.714						
18	.052	.286	100.000						
Extraction method: Principal component analysis.									

Supplemental Item 2b: Principal Component Analysis (Rotated component matrix).

Factor solution	1	2	3	4	5	6	7
age	-.057	-.090	-.122	-.173	**.667**	**.548**	-.025
mRS before admission	**.587**	**.260**	**-.362**	**-.408**	.019	**.218**	**-.230**
NIHSS on admission	**.216**	.103	.117	.111	-.070	**.866**	-.071
BP systolic on admission	-.123	.156	**-.354**	.017	**.430**	-.020	**.634**
BP diastolic on admission	-.126	-.107	.115	-.059	.011	-.087	**.919**
Heart rate on admission	.007	-.147	.000	-.158	**.679**	**-.227**	**.359**
bmi	**.894**	-.036	-.038	-.129	.022	.220	-.194
IVtPA dosis	**.828**	**-.222**	-.134	.150	-.160	.173	.128
Numberofpassages	.050	**.805**	.073	-.137	-.023	.036	-.170
NIHSS24hFU	**.230**	**.318**	-.107	**-.293**	.019	**.691**	-.068
NIHSSdischarge	**.383**	**.563**	-.089	-.163	**.246**	**.344**	**-.349**
TICI_after_Intervention	**-.767**	**-.393**	.062	.110	-.080	.033	.136
mean_postap_max	-.105	.031	**.946**	.021	-.076	-.022	.005
mean_postlat_max	-.042	-.179	.047	**.888**	-.131	-.043	-.133
mean_postap_maxslope	-.139	.049	**.919**	.049	.194	.038	-.011
mean_postlat_maxslope	-.078	.138	.011	**.916**	.088	-.041	.056
mean_postap_ttp	-.018	**-.269**	**-.231**	-.181	**-.786**	-.015	.052
mean_postlat_ttp	.055	**-.816**	-.018	-.169	-.051	-.189	**-.227**

Extraction method: Prinicpal component analysis.

Rotation method: Varimax with Kaiser normalization.

Rotation converged after 7 iterations.

Supplemental Item 3: Comparison of Real NIHSS Values and Regression-Based NIHSS Predictions Post-Thrombectomy. The comparison involves the real NIHSS values assessed 24 hours after mechanical thrombectomy (**NIHSS24h**) and at discharge (**NIHSSdc**) with the NIHSS values derived from regression equations (from figure 4 and 5). Relating to figure 4 and 5, **"DSA-based"** refers to models a, while **"TICI-based"** refers to models b. Models c and d, incorporating the "Time from Symptom Recognition to Flow Restoration (or **Tflow**)", are represented as **"DSA-based with T_{flow}"** and **"TICI-based with T_{flow}."** The standard deviations (SD) for the entire regression equations used to predict the NIHSS were derived from Figures 4 and 5. The **interrater correlation (ICC)** was calculated for the real NIHSS values and the NIHSS values derived from DSA-based and TICI-based regression models (at the end of the table). In cases of missing or not available data (n/a), the remaining data were in some cases sufficient enough to estimate a potential NIHSS value.

Patient-ID	NIHSS24h					NIHSSdc				
	Real Data	DSA-based (SD ± 4.3)	DSA-Based with T_{flow} (SD ± 4.9)	TICI-based (SD ± 4.2)	TICI-based with T_{flow} (SD ± 4.8)	Real Data	DSA-based (SD ± 2.9)	DSA-Based with T_{flow} (SD ± 2.9)	TICI-based (SD ± 3.4)	TICI-based with T_{flow} (SD ± 3.4)
1	n/a					n/a				
2	10	13.51	14.18	14.01	14.49	n/a	11.04	10.99	8.93	9.12
3	18	15.93		18.69		n/a	14.75		16.62	16.23
4	17	11.82	10.98	11.89	12.89	19	6.39	9.42	7.69	7.32
5	11	10.83		13.65		n/a	7.18		14.14	12.63
6	11	8.38	9.97	8.55	8.48	n/a	6.48	6.25	4.92	4.65
7	n/a	4.84	1.08	4.86	1.61	2	3.28	1.20	4.90	3.27
8	25	17.73	16.25	16.11	18.3	0	8.05	8.21	8.64	10.05
9	18	11.33	12.19	10.04	10.8	2	7.15	7.90	5.85	6.00
10	12	16.91	16.36	15.9	15.66	5	11.21	10.99	7.71	8.70
11	18	18.77	22.72	19.67	26.89	17	14.26	11.29	12.03	13.62
12	8	15.4	16.14	14.71	15.28	4	9.37	10.33	7.40	8.25
13	12	11.62	11.85	8.87	9.19	12	10.22	8.45	7.07	6.42
14	18	16.19	15.79	14.48	15.34	9	11.69	10.01	7.09	7.80
15	12	15.05	9.82	12.68	11.69	13	9.32	4.97	8.00	7.77
16	15	14.09	14.23	15.04	15.54	12	11.05	10.05	11.39	11.34
17	4	11.17		9.66		1	7.23		5.23	5.10
18	16	10.91	12.23	12.81	15.23	13	7.26	7.81	8.93	9.12

Patient-ID	NIHSS24h					NIHSSdc				
	Real Data	DSA-based (SD ± 4.3)	DSA-Based with T_{flow} (SD ± 4.9)	TICI-based (SD ± 4.2)	TICI-based with T_{flow} (SD ± 4.8)	Real Data	DSA-based (SD ± 2.9)	DSA-Based with T_{flow} (SD ± 2.9)	TICI-based (SD ± 3.4)	TICI-based with T_{flow} (SD ± 3.4)
19	11	12.27	15.04	12.4	16.01	6	7.01	7.88	9.24	9.57
20	10	14.19	10.27	11.88	10.76	5	6.99	5.87	5.85	6.00
21	12	13.91	12.3	14.31	13.53	2	7.71	10.05	9.55	10.02
22	16	11.62	15.22	15.41	16.37	16	8.40	9.62	10.17	10.92
23	2	6.86		5.7		1	5.11		3.99	3.30
24	1	7.79	7.29	14.64	14.6	1	4.11	4.26	11.39	11.34
25	1	8.71	5.75	7.67	5.86	1	4.70	2.33	4.92	4.65
26	13	9.92	7.42	7.95	8.18	0	6.32	3.52	3.68	2.85
27	12	5.48	4.17	7.59	6.88	7	2.98	-.01	4.92	4.65
28	12	5.26	3.28	9.91	13.51	12		-4.26	7.07	6.42
29	11	12.74	11.47	12.05	12.45	9	7.08	7.79	7.69	7.32
30	5	10.08	7.02	11	12.38	5	3.31	3.67	5.85	6.00
31	4	5.73	4.46	6.21	4.16	1	4.62	2.38	3.06	1.95
32	2	12.54		10.58		2	8.23		6.47	6.90
33	2	4.02	3.56	4.86	3.64	1	3.62	2.80	2.75	1.50
34	6	3.47	10.7	3.09	11.42	6	2.55	4.26	3.06	1.95
35	9	4.2	3.9	9.26	9.99	2	1.92	3.55	7.38	6.87
36	8	7.27	6.64	8.36	6.74	0	4.87	2.33	5.85	6.00
37	0	10.93	11.25	9.98	11.94	0	6.01	6.69	5.23	5.10
38	1	5.03	0.48	3.28	-0.28	1	3.19	-.53	2.13	.60
39	4	6.14		6.51		4	3.36		5.83	4.62
40	3	7.45	6.32	7.21	5.54	3	5.21	5.12	4.30	3.75
41	2	5.89	7.21	5.84	5.17	0	5.19	4.40	6.76	5.97
42	12	-0.84		1.08		12	-.76		.89	-1.20
43	19	13.1	19.22	12.56	20.45	3	8.54	11.23	9.24	9.57
44	n/a					n/a				

Patient-ID	NIHSS24h					NIHSSdc				
	Real Data	DSA-based (SD ± 4.3)	DSA-Based with T_{flow} (SD ± 4.9)	TICI-based (SD ± 4.2)	TICI-based with T_{flow} (SD ± 4.8)	Real Data	DSA-based (SD ± 2.9)	DSA-Based with T_{flow} (SD ± 2.9)	TICI-based (SD ± 3.4)	TICI-based with T_{flow} (SD ± 3.4)
45	n/a					n/a				
46	19	16.51		13.13		n/a	11.56		6.78	7.35
47	n/a					n/a				
48	28	20.42	22.67	19.11	22.73	n/a	12.46	14.52	12.03	13.62
49	6					2				
50	3					3				
51	12	14.77		16.73		n/a	10.59		15.38	14.43
52	15	16.8		16.49		12	10.30		11.41	12.72
53	21	15.38	22.61	13.73	20.38	16	9.83	14.58	8.02	9.15
54	14	11.98	12.41	12.59	13.72	15	8.27	8.48	6.16	6.45
55	11	12.03	10.3	12.2	12.57	12	6.21	5.06	8.00	7.77
56	22	15.63		18.21		21	16.03		16.62	16.23
57	14	16.85	13.37	14.8	13.92	4	13.74	8.49	7.09	7.80
58	11	16.66	12.77	14.8	14.14	5	7.96	7.05	7.09	7.80
59	10	12.62	12.75	12.82	14.46	2	6.78	7.47	6.47	6.90
60	11	11.73		13.89		11	11.23		14.14	12.63
61	8	13.11	11.32	10.77	11.38	0	8.07	6.17	5.54	5.55
62	14	11.74	11.41	11.55	9.99	12	8.01	7.65	8.31	8.22
63	17	14.38	15.87	14.69	16.22	10	8.40	8.74	8.02	9.15
64	16	14.71	17.61	14.09	17.92	18	8.79	11.15	11.08	10.89
65	18	14.55		13.14		10	7.91		6.47	6.90
66	14	14.53	16.33	11.13	11.92	3	9.57	10.80	6.78	7.35
67	18	12.61	15.45	12.66	14.53	14	8.54	11.43	8.62	8.67
68	20	13.8		12.97		14	8.48		6.78	7.35
69	7	7.64	9.9	11.63	13.86	7	4.62	8.77	6.16	6.45
70	2	8.09	5.29	6.01	5.22	0	3.99	.77	4.30	3.75

Patient-ID	NIHSS24h					NIHSSdc				
	Real Data	DSA-based (SD ± 4.3)	DSA-Based with T_{flow} (SD ± 4.9)	TICI-based (SD ± 4.2)	TICI-based with T_{flow} (SD ± 4.8)	Real Data	DSA-based (SD ± 2.9)	DSA-Based with T_{flow} (SD ± 2.9)	TICI-based (SD ± 3.4)	TICI-based with T_{flow} (SD ± 3.4)
71	7	12.32	9.7	12.18	11.98	7	5.84	3.95	6.47	6.90
72	4	7.68	4.4	6.19	5.16	0	3.75	.52	3.68	2.85
73	1	3.11	1.18	3.15	2.26	1	2.36	-.77	1.20	-.75
74	6	11.58	7.58	8.62	8.02	1	5.09	3.03	5.23	5.10
75	1	2.85	2.05	4.16	4.58	1	1.84	-.74	2.13	.60
76	5	10.01	9.07	10.86	12.69	1	4.72	3.84	7.38	6.87
77	5	6.91	8.45	8.97	9.48	2	5.33	6.68	4.30	3.75
78	9	8.08	7.08	7.48	6.66	5	8.15	5.15	3.37	2.40
79	5	10.97	7.97	9.98	9.06	1	5.55	3.61	5.23	5.10
80	17	7.17	11.62	5.2	14.48	2	4.21	4.55	2.13	.60
81	3	9.05	8.16	8.82	9.35	0	5.32	5.35	6.14	5.07
82	1	6.7	0.85	4.1	0.98	1	3.38	-.01	1.51	-.30
83	3	15.2		15.58		1	9.80		9.86	10.47
84	1	9.97	9.16	9.25	8.88	0	5.90	4.67	5.54	5.55
85	2	0.82		6.36		0	4.08		3.37	2.40
86	9	14.85	13.52	11.55	11.42	4	11.09	9.04	6.16	6.45
87	0	3.43	6.81	7.68	6.16	0	5.34	4.72	4.61	4.20
88	17	10.13	10	11.65	13.16	17	5.33	4.67	5.54	5.55
89	19	13.89	13.02	14.47	13.99	19	8.01	8.46	9.55	10.02
90	6	11.94	15.97	15.25	16.1	4	8.87	9.26	8.02	9.15
Interrater Correlation Coefficient with Real Data		0.738	0.838	0.716	0.817		0.55	0.6	0.6	0.63
p-Value		<0.001								
